# A novel prognostic scoring system combining the revised Tokuhashi score and the New England spinal metastasis score for preoperative evaluation of spinal metastases

**DOI:** 10.3389/fsurg.2024.1349586

**Published:** 2024-03-05

**Authors:** Dionisia Mavritsakis, Louis-Philippe Amiot

**Affiliations:** ^1^ Royal College of Surgeons in Ireland, Dublin, Ireland; ^2^ The Department of Orthopedic Surgery-Spine, Hôpital Maisonneuve-Rosemont, Montréal, QC, Canada

**Keywords:** Tokuhashi, spine, orthopedic surgery, NESMS, score

## Abstract

**Purpose:**

Numerous scoring systems have been developed in order to determine the prognosis of spinal metastases. Predicting as accurately as possible the life expectancy of patients with spinal metastatic disease is very important, as it's the decisive factor in selecting the optimal treatment for the patient. The Revised Tokuhashi score (RTS) and the New England Spinal Metastasis score (NESMS) are popular scoring systems used to determine the optimal treatment modality. However, they sometimes provide conflicting results. We propose a novel prognostic scoring system, which combines the RTS and NESMS scores in order to predict with greater accuracy the prognosis.

**Methods:**

We retrospectively reviewed the data of 64 patients with spinal metastasis enrolled between 2012 and 2021 in the Department of Orthopedic Surgery-Spine, Hôpital Maisonneuve-Rosemont, Montréal, Que. The new score per patient was calculated as a combination of the RTS of each patient and the patient's corresponding NESMS. The new score was then compared to the actual patient survival period and divided into 3 categories: Low, Moderate and Good prognosis. We then compared the accuracy of our new score to RTS.

**Results:**

In the Low Prognosis group, the reliability of predicting the prognosis was 51.9% in 27 patients. In the Moderate Prognosis group, the reliability of predicting the prognosis was 95.8% in 24 patients. In the Good Prognosis group, the reliability of predicting the prognosis was 100% in 13 patients. Our new score was found more accurate than RTS as the R2 parameter corresponding to the new score was significantly increased compared to the same parameter corresponding to the RTS score indicating a higher percentage of survival predictability for the new score as compared to the RTS score.

**Conclusion:**

This study demonstrates that a new prognostic scoring system, which would combine the RTS and the NESMS, is promising in providing an improved accuracy for predicting the actual patient survival, especially for the moderate and good prognosis patients. An appropriate prospective investigation with a larger sample size should be conducted in order to further investigate the validity of this novel scoring system and its overall predictive value.

## Introduction

The present study proposes a novel scoring system for determining the survival in patients with spinal metastasis. It is based on two already validated scoring systems, namely the Revised Tokuhashi Score (1–3) and the New England Spinal Metastasis Score (4, 5). This combined scoring system could perhaps be a better predictor of survival prognosis, thereby providing better guidance when deciding whether a patient afflicted with spinal metastasis can benefit from spinal surgery for metastatic spinal tumors.

Tokuhashi et al. (6) (2014) analysed six prognostic systems including Bauer score (7, 8), Katagiri score (9), Linden score (10), Rades score (11), Tokuhashi score (1–3, 6, 12) and Tomita score (13–15). Each of these scoring systems differ as they use a different combination of factors affecting prognosis. Two common factors in these prognostic scoring systems are the primary site of cancer and visceral metastasis, but other factors differ in each of these systems. Tokuhashi et al. (6) (2014) reviewed these scoring systems and described the significance and various limitations of these scoring systems. All of the reviewed scores were considered useful only to roughly predict the survival period. In addition, even if these scores were used to decide on the operative indications and avoidance of excessive medical treatment, it was acknowledged that future research would be needed, such as to include more oncological viewpoints with adjustment of the process of treatment (6). Aoude et al. (16) (2018) stated that the revised Tokuhashi score may be used to estimate actual patient survivorship with possible modifications.

As an alternative approach, Schoenfeld et al. (4, 5) proposed a different scoring system, the New England Spinal Metastasis Score that was focused on the 1-year mortality probability, as a primary outcome. The 6-month mortality and the overall mortality, at any point from patient enrollment in the study, were also secondary outcomes. Their cohort of patients were prospectively followed to one of two predetermined study end points: death, or survival at 365 days following enrollment or longer. While strong agreement was reported between the predicted survival vs. the observed survival, the NESMS system does not attempt to predict the patient survival beyond the 1-year period after enrollment (4).

Since cancer treatments are evolving, it is relevant to review and refine such scoring systems as prognoses may improve for the same pathology. The goal of this study was to calculate a new score for the prediction of metastatic spinal tumor outcome by combining two existing validated scoring systems, namely RTS and NESMS. By combining these two proven scoring systems, the new combined score could potentially be more accurate at predicting metastatic spinal tumor prognosis as the combined score would consider a wider variety of factors that influence the prognosis.

## Methods

This study was conducted retrospectively at the The Department of Orthopedic Surgery-Spine, Hôpital Maisonneuve-Rosemont, Montréal, QC, Canada, which serves a broad uptake area including the metropolitan region of Montreal and its surroundings. This referral center captures a diverse patient population, ensuring the generalizability of our findings. The study was approved by the ethics review board of The Department of Orthopedic Surgery-Spine, Hôpital Maisonneuve-Rosemont, Montréal, QC, Canada. We enlisted a total of 64 patients in this investigation. The average age of patients was recorded at 64.8, with a standard deviation SD = 13.3. The population was fairly equally distributed between males and females (31 male patients and 33 female patients). All the scores and factors involved were based on data collected at diagnosis, before a treatment was started. We considered patients with various types of cancer, including lung cancer (19 patients, 29.7%), breast cancer (16 patients, 25%), hematological cancer (11 patients, 17.2%), sarcomas (10 patients, 15.6%), and other cancers (8 patients, 12.5%).

The inclusion of patients with sarcomas, constituting 15.6% (10 out of 64) of our cohort, was a deliberate choice to ensure that our findings reflect the clinical diversity encountered in a specialized spinal surgery practice. Although sarcomas have distinct treatment paradigms, our scoring system is designed to be applicable across the broad spectrum of spinal metastatic disease, including less common etiologies such as sarcomas. This represents our effort to capture the full array of conditions treated in such settings. The inclusion of this subgroup, which is a substantial portion of our study population, reinforces the versatility and clinical relevance of our prognostic tool in a real-world healthcare setting, offering valuable insights for clinicians managing a wide range of spinal metastases.

Our cohort included patients with performance statuses ranging from poor (5 patients, 7.8%), moderate (37 patients, 57.8%), to good (22 patients, 34.4%). We observed a spectrum of extraspinal bone metastases foci; 41 patients (64.1%) had no foci, 19 (29.7%) had 1–2 foci, and 4 (6.2%) had 3 or more. The number of spinal metastases in the vertebral body ranged from none in 25 patients (39.1%), 1–2 in 14 patients (21.8%), to 3 or more in 25 patients (39.1%). Metastases to major internal organs were present and varied from nonremovable, removable, to none, with respective patient counts of 10 (15.6%), 21 (32.8%), and 33 (51.6%). Palsy was noted as complete in 3 patients (4.7%), incomplete in 58 patients (90.6%), and absent in 3 patients (4.7%). This inclusive approach to patient selection ensures that our study reflects a real-world clinical scenario, thus enhancing the external validity and applicability of our novel prognostic scoring system.

The RTS is based on six parameters: (I) general condition (performance status classified as poor, moderate or good) (II) the number of extra spinal bone metastases foci, (III) the number of spinal metastases in the vertebral body (IV) the number of metastases to the major internal organs (V) the primary site of cancer, and (VI) the presence of spinal cord palsy (1) (Table 1). Each parameter is given a rating between 0 and 2 except for primary tumor, which is given a rating between (0 to 5) depending on the primary site of the cancer. The life expectancy is then predicted by classifying the calculated RTS. A total RTS of 0–8 has a mean life expectancy of less than 6 months, a total RTS of 9–11 has a mean life expectancy of greater than or equal to 6 months and a total RTS of 12–15 has a mean life expectancy of greater than or equal to one year (1) (Table 2).

**Table 1:** Revised Tokuhashi evaluation system—score parameters, points, and percentage of patients.

Tokuhashi score parameters	Ratings (points)	No. (%) of patients
General condition (performance status)		
Poor (PS 10–40%)	0	5 (7.8)
Moderate (PS 50–70%)	1	37 (57.8)
Good (PS 80–100%)	2	22 (34.4)
No. of extraspinal bone metastases foci		
≥3	0	4 (6.2)
1–2	1	19 (29.7)
0	2	41 (64.1)
No. of spinal metastases in the vertebral body		
≥3	0	25 (39.1)
1–2	1	14 (21.8)
0	2	25 (39.1)
Metastases to the major internal organs		
Nonremovable	0	10 (15.6)
Removable	1	21 (32.8)
None	2	33 (51.6)
Primary site of the cancer		
Lung, osteosarcoma, stomach, bladder, oesophagus, pancreas	0	27 (42.2)
Liver, gallbladder, unidentified	1	2 (3.1)
Others	2	0
Kidney, uterus	3	18 (28.1)
Rectum	4	2 (3.1)
Thyroid, breast, prostate, carcinoid tumour	5	15 (23.4)
Palsy		
Complete (Frankel A, B)	0	3 (4.7)
Incomplete (Frankel C, D)	1	58 (90.6)
None (Frankel E)	2	3 (4.7)

**Table 2:** Life expectancy predicted by the RTS (1).

RTS score	Mean life expectancy
	(in months)
Low Prognosis: 0–8	<6 months
Moderate Prognosis: 9–11	≥6 months
Good Prognosis: 12–15	≥1 year

RTS, revised Tokuhashi score.

The predicted prognosis of a patient based on the RTS is presented in Table 2. Once the patient's survival period is determined, using the RTS score, treatment strategies are selected which can include palliative care, no surgery, spinal decompression or even wide or marginal excision of spinal tumors (17).

In contrast to the RTS, the NESMS does not attempt to estimate the patient's expected survival after diagnosis. Instead, the NESMS is a tool intended to predict the patient's mortality probability for only up to one year after enrollment/diagnosis, as a primary outcome, and up to 6 months or any time during enrollment, as a secondary outcome (4). The NESMS takes into account the factors summarized in Table 3, which include the Modified Bauer Score, the ambulatory function of the patient and the value for serum albumin in the blood. The mortality percentages associated to the NESMS are outlined in Table 4. The NESMS system assigns a score to each patient ranging from 0 to 3. A higher NESMS score is associated with superior survival following treatment. The two extreme values, 0 and 3, correspond respectively to the two predetermined study end-points: death, or survival at 365 days following enrollment or longer. Schoenfeld *et al*. (4) validated the NESMS evidence-based scoring system and demonstrated its accuracy in determining survival in patients with spinal metastasis.

**Table 3:** Description of the New England spinal metastasis score (4).

NESMS Characteristic	Points Assigned
1. Modified Bauer Score	
* No visceral metastases (1 point)*	*-*
* Primary tumor is not lung cancer (1 point)*	*-*
* Primary tumor is breast, renal, lymphoma,or myeloma (1 point)*	*-*
* Single skeletal metastasis (1 point)*	*-*
Modified Bauer Score ≤ 2	0
Modified Bauer Score ≥ 3	2
2. Ambulatory function	
Dependent ambulator/nonambulatory	0
Independent ambulator	1
3. Serum Albumin	
<3.5 g/dl	0
≥3.5 g/dl	1

**Table 4:** Patient mortality percentages at 6-month, 1-year and overall, by NESMS designation (4).

NESMS	6-month mortality (%)	1-year mortality (%)	Overall mortality (%)
0	85	100	100
1	63	78	83
2	27	48	60
3	10	15	30

Although these two scoring systems are not directly comparable, as they focus on different outcomes, it was reasonable to expect, a certain level of correlation between the RTS and the NESMS values for a certain patient. As such for our sample of patients, both score values for each patient were calculated and compared in order to determine if there was agreement on the expected survival projected by each scoring system. While a limited correlation between the two scores was observed, it was also noted that the NESMS values were scattered throughout the cohort of patients, and were not necessarily in agreement with the RTS values. For example, there were patients with low RTS scores (RTS < 8), indicating an expected survival of up to six more months, but with high NESMS score values (NESMS = 2 or 3), indicating a higher probability of survival for up to one more year, at least. The opposite situation was also observed for other patients. These conflicting projections between the two scoring systems about the expected patient survival period, prompted us to determine a method that would reasonably combine these two well-established scoring systems, in an attempt to improve the accuracy of the expected survival for a particular patient. After careful consideration, a process to combine these two scoring systems was determined which consisted of adding up to two score points to the RTS score, for patients with higher NESMS, and subtracting up to two score points from the RTS, for patients with lower NESMS (Table 5).

**Table 5:** New score—combining RTS and NESMS.

Calculated NESMS score	Combined RTS & NESMS score
0	RTS score—2 points
1	RTS score—1 point
2	RTS score + 1 point
3	RTS score + 2 points

NESMS, New England spinal metastasis score; RTS, revised Tokuhashi score.

## Results and discussion

The novel scoring system that is presented in this study is based on combining the two already validated scoring systems, namely the Revised Tokuhashi Score (1) and the New England Spinal Metastasis Score (4, 5). For the new combined score, it was decided to keep the calculating frame of the RTS score with the same group categories. After the RTS score was determined for a particular patient, the following adjustments were made to the RTS in order to determine the new combined score. If the NESMS was equal to zero, the patient's RTS was reduced by two points. If the NESMS was equal to one, the patient's RTS was reduced by one point. If the NESMS was equal to two, the patient's RTS was increased by one point. If the NESMS was equal to three, the patient's RTS was increased by two points (Table 5). As such, the upper limit determined for the new score is 17 which is the maximum RTS score (RTS = 15) plus two points which would be added if the NESMS was equal to 3, the highest possible score for this scoring mechanism. Consequently, the patients were divided into three categories: Low prognosis group (New score 0–8, number of patients *n* = 27), Moderate prognosis group (New score 9–12, *n* = 24) and Good prognosis group (New score 13–17, *n* = 13). The score limits of these categories are based on the RTS categories, which were slightly modified in order to reflect the higher maximum score limit of this new scoring system.

Patients with a total new score of 8 or less were included in the low prognosis group. The low prognosis group has a predicted survival period of less than 6 months. Twenty-seven patients out of the 64 identified patients with spinal metastases were classified in the low prognosis group based on the new scoring system. Out of the 27 patients, 14 patients effectively survived less than 6 months. As such, in the low prognosis group, the new scoring system accurately predicted the survival period for fourteen patients out of the twenty-seven in this group, which correlates to a 51.85% accuracy for the predicted prognosis of the new score (Table 6).

**Table 6:** Novel score combining RTS and NESMS score correlated to actual survival period.

Category	Novel score	Predicted prognosis	Actual number of patients	Accurate category classifictaion	Accuracy (%)
Low prognosis group	0–8	<6 months	27	14	51.9%
Moderate prognosis group	9–12	≥6 months	24	23	95.8%
Good prognosis group	13–17	≥1 year	13	13	100.0%

Patients with a total new score ranging between 9 and 12 were included in the moderate prognosis group. The moderate prognosis group has a predicted survival period of 6 months or more. Twenty-three patients out of the 64 identified patients with spinal metastases were classified in the moderate prognosis group based on the new scoring system. Out of the 24 patients, 23 patients effectively survived 6 months or more. As such, in the moderate prognosis group, the new scoring system accurately predicted the survival period for twenty patients out of the twenty-one in this group, which correlates to a 95.8% accuracy for the predicted prognosis of the new score (Table 6).

Patients with a total new score ranging between 13 and 17 were included in the good prognosis group. The good prognosis group has a predicted survival period of 12 months or more. Thirteen patients out of the 64 identified patients with spinal metastases were classified in the good prognosis group based on the new scoring system. All of the thirteen patients effectively survived 12 months or more. As such, in the good prognosis group, the new scoring system accurately predicted the survival period for all patients in this group, which correlates to a 100% accuracy for the predicted prognosis of the new score (Table 6).

**Figure 1:**
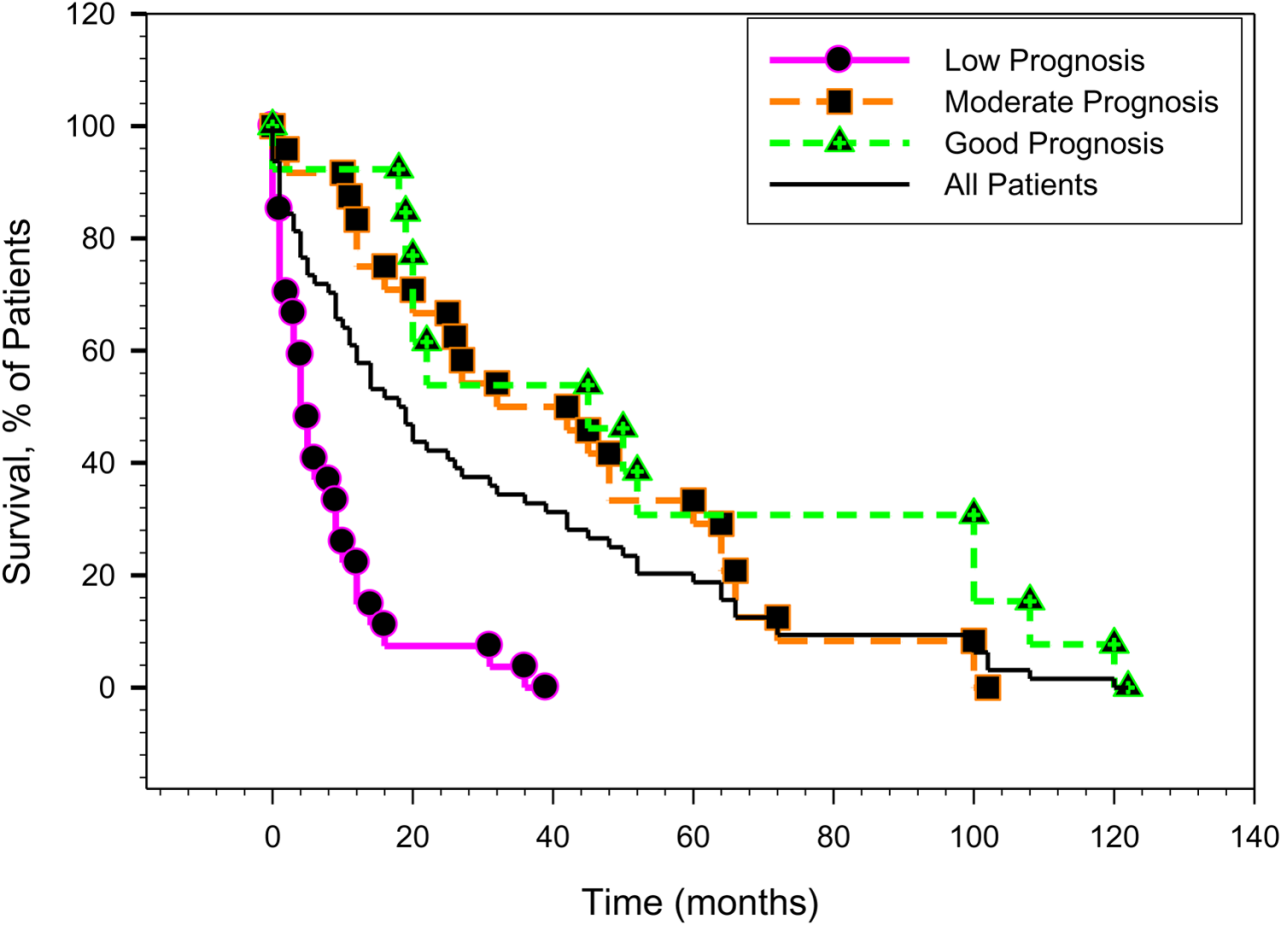
The kaplan-meier survival plots as a function of time, corresponding to the new score.

The patients actual survivals are also graphically presented in Figure 1, which shows the Kaplan-Meier survival curves, based on our new score prognosis categories (low, moderate and good prognosis).

**Figure 2:**
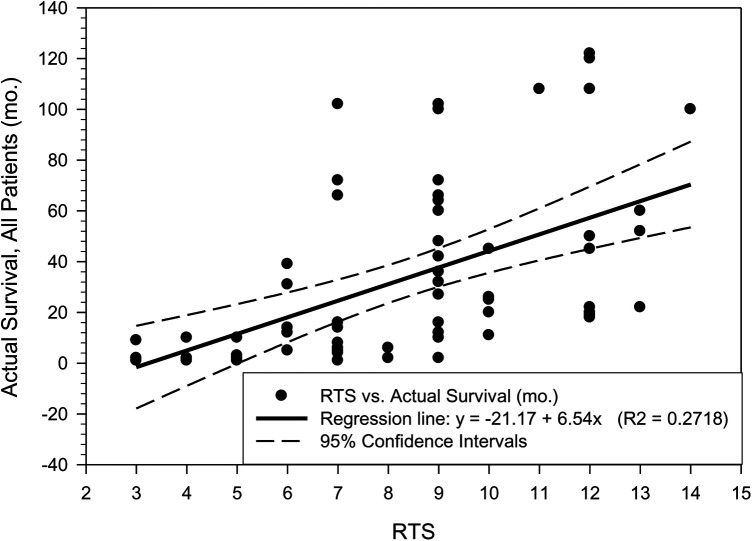
Patient actual survival (mo.) vs. the RTS score (R2 = 0.2718).

**Figure 3:**
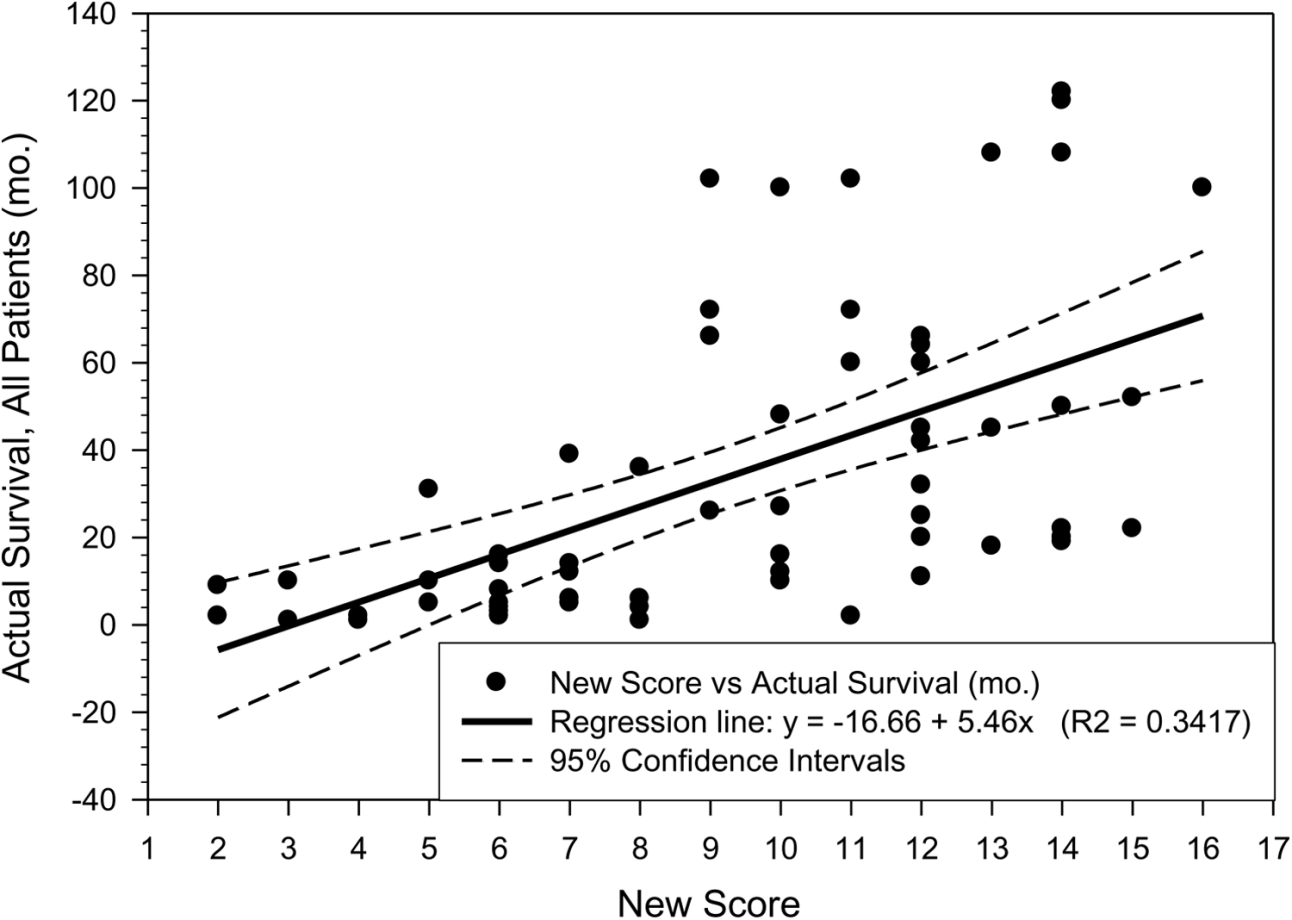
Patient actual survival (mo.) vs. the New Score (R2 = 0.3417).

The patient survival in the moderate and good prognosis categories is very similar which may be explained by the limited number of patient data available for this study and also by the incremental improvements in the treatment options made available to patients in the last 15 to 20 years. The plots “RTS vs. Actual Patient Survival”, as well as the “New Score vs. Actual Patient Survival” are shown in Figures 2, 3. While there are similarities between the two plots, the R2 parameter corresponding to the new score (Figure 3) is significantly increased compared to the same parameter corresponding to the RTS score (Figure 2), indicating a higher percentage of survival predictability for the new score as compared to the RTS score. Specifically, in Figure 3, 34.17% of the patient survival can actually be predicted by the new score model, vs. a 27.18% prediction percentage in the case of the RTS model (Figure 2). We believe that this is an important accuracy improvement in our new score model when compared to the RTS model, perhaps even more significant than the percentages indicated in Table 6. For reference purposes, the limits of the 95% confidence intervals are also depicted in Figures 2, 3, with the margins of error noticeably smaller in Figure 3, as compared to those in Figure 2. The limited number of patients in our cohort prevented us to investigate the patient survival for each specific type of cancer. Additional research will be needed in the future, as new data becomes available, not only to validate the results presented in this work, but also to explore the survival rate for each particular type of cancer.

### Integrating quality of life in prognostic models

There is a traditional focus on life expectancy in treatment selection for spinal metastatic disease, however, Quality of Life (QoL) is also critically important in determining the optimal treatment pathway. Recent research increasingly underscores the need to balance predicted survival with the patient's functional status, pain management, psychological well-being, and overall satisfaction with life. While our current scoring system offers a novel approach to predicting survival, it does not directly address these QoL dimensions. Future iterations of this scoring system could integrate specific QoL metrics, such as the EORTC QLQ-C30 questionnaire or the SF-36 health survey. This integration could provide clinicians with a more comprehensive tool for treatment decision-making, ultimately aiming to enhance both the length and quality of life for patients with spinal metastatic disease.

### Strengths, limitations, and clinical implications

This study's primary strength lies in its novel approach of integrating the Revised Tokuhashi Score with the New England Spinal Metastasis Score, aiming to enhance prognostic accuracy for spinal metastasis. The combination of these two established scoring systems is a testament to our commitment to evolving the prognostic tools to better reflect the nuances of modern clinical practices. Regarding limitations, we recognize that our study, given its retrospective nature and reliance on data from a single center, may have certain constraints in terms of generalizability. The modest cohort size of 64 patients, particularly the “Good Prognosis” subgroup, while reflective of the specific patient population treated during the study period, introduces considerations about the statistical power of our findings. This sample size, though deliberate and representative of our clinical setting, means our results should be viewed as indicative and preliminary. They lay the groundwork for future studies but may not have the statistical power typically desired for broader generalization.

Moreover, the inclusion of a substantial proportion of sarcoma patients (15.6%) may influence the generalizability of our findings to broader oncological populations. Despite these limitations, our study contributes significantly to the field by offering a simplified, point-based prognostic system. This system may be particularly advantageous in clinical settings where extensive prognostic models are not feasible, providing a practical tool to assist in initial clinical decision-making processes. While it does not replace comprehensive clinical judgment, it serves to augment it, especially in resource-constrained environments. Looking ahead, we advocate for further research involving larger, multi-center cohorts to validate and refine our scoring system, ensuring its robustness and applicability across diverse clinical landscapes.

### Future directions and the role of AI and machine learning

In the realm of medical prognostics, particularly for spinal metastasis, we are witnessing a paradigm shift driven by technological advancements and a deeper understanding of disease complexity. Our combined scoring system, integrating the Revised Tokuhashi Score and the New England Spinal Metastasis Score, marks a significant step forward. However, the true potential of prognostic modeling may be realized through a two-pronged approach: the granular integration of the most salient parameters from existing systems and the application of AI and machine learning technologies. The synthesis of key parameters from established scoring systems into a cohesive model promises a more personalized and precise prognostication. This tailored approach aligns with the principles of personalized medicine, ensuring that each patient's unique clinical profile is reflected in their prognostic assessment.

Simultaneously, AI and machine learning stand to revolutionize prognostic modeling. These technologies offer the ability to assimilate a broader range of variables, including intricate clinical details and Quality of Life (QoL) metrics. They can discern complex patterns within large datasets, patterns that may elude traditional analytical methods. This not only enhances the predictive accuracy of our models but also allows prognostic predictions to be more dynamic, adapting and refining as new patient data becomes available.

By embracing this dual approach, integrating both a nuanced parameter-based methodology and advanced computational techniques, we can transform our scoring system into a robust, adaptive, and deeply insightful tool. This tool will not only capture the complexities of individual patient profiles but will also evolve in tandem with the rapidly advancing landscape of medical science and technology.

## Conclusion

The present work is a retrospective evaluation of the actual survival of a sample of 64 patients afflicted with spinal metastases. This study proposes a new scoring system to predict the potential survival of a patient with spinal metastatic disease at the time of diagnosis, in an attempt to provide a more appropriate treatment path for the patient. This new prognostic scoring system combines two well-established scoring systems, namely the Revised Tokuhashi Score (RTS) (1) and the New England Spinal Metastasis Score (NESMS) (4, 5), in an effort to provide improved accuracy for predicting the actual patient survival. By combining these two proven scoring systems, the new combined score could potentially be more accurate at predicting metastatic spinal tumor prognosis as the combined score considers a wider variety of factors that influence the prognosis. An appropriate prospective investigation with a larger sample size should be conducted in the future to further investigate the validity of this novel scoring system and its overall predictive value.

## Data Availability

The raw data supporting the conclusions of this article will be made available by the authors, without undue reservation.
